# The effect of patient care order sets on medical resident education: a prospective before-after study

**DOI:** 10.1186/1472-6920-13-146

**Published:** 2013-11-06

**Authors:** Catherine H Yu, Anne L Stephenson, Samir Gupta

**Affiliations:** 1 Division of Endocrinology, Department of Medicine, St. Michael’s Hospital, 30 Bond St., M5B 1 T8, Toronto, ON, Canada; 2 Keenan Research Centre in the Li Ka Shing Knowledge Institute of St. Michael's Hospital, Toronto, Canada; 3 Division of Respirology, Department of Medicine, St. Michael’s Hospital, Toronto, Canada; 4 Department of Medicine, University of Toronto, Toronto, Canada

**Keywords:** Clinical decision support systems, Medical order entry systems, Internship and residency, Medical education, Guideline adherence, Hospital information systems, Patient admission, Clinical competence

## Abstract

**Background:**

Patient care order sets are increasingly being used to optimize care. While studies have evaluated the impact of order sets on provider performance and patient outcomes, their impact on postgraduate medical trainee knowledge remains unknown. We sought to evaluate the impact of order sets on respirology knowledge, order-writing skills, and self-reported learning.

**Methods:**

We conducted a prospective before-after study. Postgraduate trainees completing a Respirology rotation at a quaternary-care hospital 6 months before (no order set period) and 12 months after (order set period) order set introduction. Guideline-based admission order sets with educational prompts detailing recommended management of cystic fibrosis and chronic obstructive pulmonary disease were implemented on the respirology ward. Each resident completed a test before and after the rotation assessing knowledge and order-writing. Residents in the order set period additionally completed a questionnaire regarding the impact of order set use on their learning. Analysis: The primary outcome, the difference between pre and post rotation scores was compared between residents in the no order set period and residents in the order set period, using univariate linear regression. Test validity was assessed with a 2-sample t-test, analysis of variance and Pearson’s correlation coefficient. Self-reported impact of order set use were descriptively analyzed, and written responses were collated and coded.

**Results:**

Investigators consecutively recruited 11 subjects before and 28 subjects after order set implementation. Residents in the order set period had a greater improvement in post-rotation test scores than residents in the no order set period (p = 0.04); after adjustment for baseline scores, this was not significant (p = 0.3). The questionnaire demonstrated excellent convergent, discriminant and construct validity. Residents reported that order sets improved their knowledge and skills and provided a systematic approach to care.

**Conclusions:**

Order sets do not appear to impair resident education, and may impart a benefit. This will require validation in larger studies and across diseases.

## Background

Patient care order sets present physicians with a template for care orders based on best evidence [1]. These decision support tools may bridge care gaps and improve quality by providing point-of-care education for providers [2,3]. As such, order sets serve primarily as patient care tools, however they may also be a vehicle for quality improvement, and potentially, an educational tool for medical trainees. While studies have evaluated the impact of order sets on provider performance and patient outcomes [4,5], their impact on postgraduate medical trainee (resident) knowledge remains unknown. Printed or computerized order sets may convey new knowledge to physicians or reinforce existing knowledge. Alternatively, order sets may serve as a barrier to knowledge acquisition by providers: by listing appropriate patient care interventions, order sets may obviate the need for physicians to seek out and learn appropriate interventions on their own. In this, way, order sets may prevent “reflection in action”, a fundamental component in reflective practice [6]. A majority of residents who use order sets perceive that they improve patient care and reduce medical errors [7]. In a previous study, order sets also increased residents’ comfort level with symptom management in palliative care [8]. In the undergraduate setting, pneumonia admission order-writing skills among medical students trained at hospitals using computerized provider order-entry (CPOE) compared to those using handwritten orders were no different [9]. However, the effect of order sets on resident knowledge and order-writing skills has not previously been reported. We sought to determine the impact of admission order sets on knowledge and order-writing skills, and their perceived utility among residents.

## Methods

### Study design

This was a prospective before-and-after exploratory study conducted on the Respirology ward of a quaternary care University of Toronto-affiliated hospital.

### Participants

We recruited participants from April 2009 to November 2010. Order sets were introduced in November 2009. Thus, the no order set period was between April and October 2009 (6 months) and the order set period was between November 2009 and November 2010 (12 months). All postgraduate medical trainees completing their one-month Respirology rotation during these time periods were recruited consecutively on the first day of their Respirology rotation by CHY. This included residents in General Internal Medicine, Anaesthesia, Radiology and Respirology, in any year of training (post-graduate years 1 to 5). Consenting residents completed knowledge tests on cystic fibrosis (CF) and chronic obstructive pulmonary disease (COPD) on day 1 prior to the rotation and the same test one month later upon completion of the rotation (Additional file 1: post-rotation questionnaire).

### Intervention

CF and COPD patient care order sets were developed by a multidisciplinary team including pulmonologists, internists, and allied health staff. Content was based on best evidence where available, and expert opinion and existing practice where evidence was unavailable. The order sets provided comprehensive admission instructions, including indication of infection control precaution requirements, monitoring, diet, activity level, multidisciplinary team referrals, respiratory care requirements (e.g. positive pressure ventilation requirements, oxygen titration instructions, and inhaled medication or disease self-management education requirements), investigations, and medications. In addition, where a strong level of evidence existed to direct practice, specific physician prompts were integrated into the order sets, with an indication of the level of evidence supporting the recommendation (as graded by clinical practice guidelines [10]) as well as the expected outcome. Although their primary purpose was to enable care, as such, the order sets could serve as an educational tool with the provision of diagnostic and management choices, evidence-based recommendations and reference resources.

In November 2009, printed order sets were developed and placed among other order sets on the ward and emergency room. In March 2010, these were converted into an identical electronic format and integrated into a new CPOE system. All residents were informed about the existence and location of printed and electronic order sets.

### Main measures

Primary outcome was the change in test score from before and after the rotation in residents in the no order set period, compared to the change in test score in residents in the order set period. Secondary outcomes included order set use, test validity and qualitative data regarding perceived impact on skills and knowledge.

### Data collection instruments

Participants completed a test, as well as a questionnaire regarding sociodemographic characteristics and clinical experience. Pre- and post-rotation tests contained identical questions, consisting of skill-testing (order-writing) and knowledge-testing (true/false and short answer questions) sections. While pre-rotation test completion may have impacted post-rotation knowledge, both no order set and order set residents would have been subject to this effect. The knowledge section tested: 1) knowledge transmitted directly by order set evidence-based prompts (for example, the COPD order set explicitly listed criteria for early use of non-invasive positive pressure ventilation; a test question asking for these criteria was classified as a type 1 question); 2) other knowledge conferred through order set use (for example, the CF order set included a section listing “GI medications” such as omeprazole, ranitidine, domperidone, ursodeoxycholic acid, polyethylene glycol and mineral oil; a test question asking for gastrointestinal consequences of CF and a corresponding pharmacotherapeutic agent for each was classified as a type 2 question); and 3) knowledge that could not be gained through order set use (for example, a question asking what percent of exacerbations of COPD are due to respiratory infections was classified as a type 3 question, as this content was not included in the order set) (question types 1,2, and 3) (Additional file 1: questionnaire scoring scheme and classification of question types). Tests and grading schemes were developed by CHY and SG and reviewed and revised by AS (content experts) before study launch.

We also included questions regarding their age, gender, stage and program of training, and prior clinical experience with patients with COPD and CF.

For residents in the order set period, we included open-ended questions as part of their post-rotation test; these questions ascertained involvement in patient care and order set use, and probed the perceived impact of order set use on knowledge and skills (Part A Questions 4 and 5, Additional file 1: post-rotation questionnaire).

### Analysis

Data are expressed as proportions, means and standard deviations. The primary outcome, change (i.e. individual improvement) in test scores from before to after the rotation, was compared between residents in the no order set period and residents in the order set period using univariate linear regression. We also tested for differences by test section and question type. We created a multivariable model with baseline scores included as a predictor variable to ensure that any observed difference in change in test score from before to after the rotation between residents in the no order set period and residents in the order set period was not a function of chance differences in baseline scores between these groups. We then re-tested any significant differences using this multivariate model including baseline scores. Because this was an exploratory study and because we used a scale that had previously not been validated, we did not calculate sample size.

For test validation, to assess convergent and discriminant validity, we used a 2-sample t-test or analysis of variance to compare baseline scores between subject types (gender, training program, year of study). To assess construct validity, we used a paired t-test to compare pre- to post-rotation scores within subjects and a Pearson’s correlation coefficient to measure correlation between baseline score and change in score.

For scoring validation, CHY and SG independently scored a random sample of 5 tests each, re-scored these in conjunction to minimize scoring variation, then independently scored remaining tests. A random sample of 20 tests was independently scored by both investigators, to measure consistency. We used a Pearson’s correlation coefficient to measure inter-rater agreement.

For qualitative analyses regarding the impact of order set use on resident knowledge, we collated and coded written responses to the open ended questions from post-rotation tests of residents in the order set period. We used SAS v9.3 for all analyses.

### Ethical approval

The study was approved by the hospital’s review board; informed consent was obtained from participants.

## Results

### Participant characteristics

Fifty-two residents rotated through the Respirology inpatient service during the study period and consented to participate in the study. However, only 39 residents (11 in the no order set group and 28 in the order set group) completed both pre-rotation and post-rotation tests; analysis for the primary outcome was conducted using this dataset. Characteristics of those who completed post-rotation tests and those who did not were similar. Age, training program, and training level distributions were similar between groups (Table 1).

**Table 1:** Demographic and training characteristics of postgraduate medical trainees in no order set and order set groups

	No order set group (n = 11) (%)*	Order set group (n = 28) (%)*
Age (years):		
25-29	7 (64%)	23 (82%)
30-34	3 (27%)	4 (14%)
>40	1 (9%)	1 (4%)
Gender:		
Female	8 (73%)	15 (54%)
Male	3 (27%)	13 (46%)
Training program:		
Internal medicine	9 (82%)	24 (86%)
Anaesthesia	1 (9%)	2 (7%)
Radiology	1 (9%)	2 (7%)
Postgraduate year:		
1	4 (36%)	6 (21%)
2	2 (18%)	14 (50%)
3	4 (36%)	7 (25%)
4	1 (9%)	1 (4%)

*As a percentage of all participants in each cohort.

### Change in test score

In both the no order set period and the order set period, individual residents’ test scores improved significantly from before to after the rotation. Residents in the order set period had a greater improvement than those in the no order set period (11 +/-8.2 vs 5.3 +/-5.5, respectively) (p = 0.04) but when adjusted for baseline score, this difference became insignificant (p = 0.3, Table 2). Similarly, greater improvements were noted in the knowledge-testing section (p = 0.03) and in Type 3 knowledge questions (p = 0.03); these were no longer significant when adjusted for baseline score (p = 0.2, p = 0.1 respectively, Table 2). In the order set group, there was no difference in score improvements in those who reported use of the order set versus those who did not (p = 0.2).

**Table 2:** Test scores in no order sets and order sets periods

	**No order set (n = 11)**	**Order set (n = 28)**	**p-value**
Pre-rotation overall test score (out of 87)	39.5 +/- 8.5	32.9 +/- 10.2	-
Post-rotation overall test score (out of 87)	44.9 +/- 9.2	43.9 +/- 7.1	-
Change in overall test score	5.3 +/- 5.5	11 +/- 8.2	p = 0.3*
Change in test score by section:			
Order-writing skill (out of 25)	1.5 +/- 2.5	3.1 +/- 3.9	p = 0.2
Knowledge (out of 62)	3.8 +/- 4.1	7.9 +/- 5.6	p = 0.2*
Change in test score by knowledge question type			
Type 1 (out of 21)	2.0 +/- 2.1	3.1 +/- 2.9	p = 0.3
Type 2 (out of 20)	1.3 +/- 1.9	2.3 +/- 3.1	p = 0.3
Type 3 (out of 21)	0.5 +/- 2.2	2.6 +/- 2.6	p = 0.1*

SD denotes standard deviation.

*multivariate analysis with baseline score included as a predictor variable.

### Order set use

Among 28 participants in the order set period, 13 (46%) used both COPD and CF order sets, 4 (14%) used the COPD order set only, 7 (25%) used the CF order set only, and 4 (14%) used neither*.* In only three instances was order set non-use attributable to the fact that the individual participant did not admit a patient with the relevant diagnosis.

### Test validity

Convergent and discriminant validity tests conducted in pre-rotation scores for all residents (n = 45) showed expected convergence by gender and discrimination by training program and year of study (Table 3). Construct validity was further supported by a strong inverse correlation between baseline score and change in score (r = -0.67, p < 0.001) (i.e. participants with lower baseline knowledge learned more), and a significant improvement in post-rotation scores across all participants [(increase by 9.38 +/- 7.91 (p < 0.0001)].

**Table 3:** Pre-rotation test scores by gender, training program and year of study

**Demographic (n)**	Pre-rotation test score*	**p-value**
Gender		p = 0.28
Male (20)	35.5 +/- 10.2	
Female (25)	35.8 +/- 9.35	
Training program		p = 0.051
Internal medicine (38)	35.5 +/- 9.16	
Non-internal medicine (7)	27.7 +/- 11.1	
Year of study (internal medicine only)		p = 0.012
1 (13)	29.0 +/- 9.04	
2 (19)	35.7 +/- 7.55	
3 (10)	41.1 +/- 9.05	

*maximum test score was 87.

### Scoring validity

There was good correlation of scoring between the scorers (R = 0.99, p < 0.0001).

### Qualitative data

Among 24 participants who used at least one order set, 14 (58%) reported that information provided in the order set stimulated further reading, 21 (88%) that usage improved knowledge, and 19 (79%) that usage improved order-writing. Two (8%) reported an impairment in knowledge and/or skill acquisition with order set use. Qualitatively, participants noted that order sets provided a systematic approach to care (particularly a patient-centred interprofessional approach) and a checkpoint for quality control, while increasing knowledge and confidence in evidence-based care. Positive quotes included: “order sets made me more systematic and organized” and “…ensured zero mistakes,” and “repetitive exposures aid memorization”. Negative quotes included “order sets turned my brain off” and “…prevented me from thinking as much…”

## Discussion

In this study, we sought to determine the impact of admission order sets on resident knowledge and demonstrated a trend towards a beneficial effect.

As in our study, Knight and colleagues previously failed to show a statistically significant effect of order sets on medical student order-writing skills [9]. However, given that only 46% of the residents in our study used both order sets during their rotation, it is possible that a larger effect would have been seen if order set uptake had been greater. Aside from the minority of residents who did not admit a patient with either COPD or CF within their 1-month rotation, possible explanations for order set non-use include a lack of awareness or availability of order sets at the point of care. Previous authors have suggested that CPOE systems can overcome this problem by “pushing” information about order set availability to the clinician in the form of a prompt at the time of order entry. This can be programmed by capturing the admission diagnosis and linking it to relevant order sets, or through an algorithmic analysis of the problem list at time of admission, to find pertinent order sets [11]. However, characteristics of the order sets themselves may also have inhibited their use. Previous studies have indicated that order set usage can vary by medical condition [12], and that certain CPOE systems can frustrate users by increasing the amount of time required for order entry [13]. Order sets designed through user-centered methodology have improved efficiency, usability, and safety [14]. Future studies should include detailed qualitative assessments of reasons for order-set use and non-use, and larger studies should attempt to identify significant predictors of order set uptake, as this may not only affect the educational impact of this intervention, but also its impact on physician behaviour patient care.

A majority of residents in the order set period reported improvements in knowledge and order-writing skills, and that they were likely to read more about the topic as a result of the order set. These qualitatively perceived benefits mirror those reported previously in other clinical settings [7,8]. However, it should also be noted that two subjects in our study believed that the order set impaired knowledge/skill acquisition. This may be congruent with previous reports suggesting that even well-designed CPOE systems can have unintended consequences among certain users, including increases in workload, disruptions in workflow, and perceived restrictions on communication [15].

Our study is limited by its small size, its before-and-after design, use of a test that had not previously been validated, conversion of paper order sets to CPOE mid-study, and suboptimal uptake of the order set itself. Strengths include use of pre- and post- rotation scores within the same subject, reducing inter-subject variability, an objective questionnaire development process, and strong measures of questionnaire construct validity. Furthermore, we used qualitative descriptions to support our quantitative findings.

## Conclusion

Order sets are becoming a ubiquitous tool for quality improvement and this study suggests that they do not appear to impair resident education, and may impart a benefit. This will require validation in larger studies with concurrent controls, across multiple centers, and across several disease-types. Finally, beneficial effects on continuing medical education and professional development among fully-trained professionals who use order sets (particularly in non-academic settings) might also be expected, and should be measured objectively in future studies. Ultimately, such research may enable order sets to assume a role in both medical education and continuing professional development educational plans and curricula.

## Abbreviations

CPOE: Computerized provider order-entry; CF: Cystic fibrosis; COPD: Chronic obstructive pulmonary disease.

## Competing interests

All authors declare no competing interest.

## Authors’ contributions

CHY participated in the study design, collected and analyzed the data, led the discussions and drafted the manuscript; she is guarantor of the manuscript. AS analyzed the data and contributed to the discussion. SG conceived of the study, analyzed the data, led the discussions and drafted the manuscript. All authors revised the manuscript critically for intellectual content, and have read and approved the final manuscript.

## Authors’ information

Catherine Yu is Assistant Professor, Division of Endocrinology, Department of Medicine and Dalla Lana School of Public Health, University of Toronto, and Associate Scientist at the Keenan Research Centre in the Li Ka Shing Knowledge Institute of St. Michael's Hospital. She is a Clinician-Educator with an interest in knowledge translation, including the role of innovative educational and care tools in improving quality of care and in reducing clinical care gaps.

Anne Stephenson is Assistant Professor, Division of Respirology, Department of Medicine, St. Michael’s Hospital, Toronto, Ontario, Canada and Associate Scientist at the Keenan Research Centre in the Li Ka Shing Knowledge Institute of St. Michael's Hospital.

Samir Gupta is Assistant Professor, Division of Respirology, Department of Medicine, St. Michael’s Hospital, Toronto, Ontario, Canada and Associate Scientist at the Keenan Research Centre in the Li Ka Shing Knowledge Institute of St. Michael's Hospital. He is a Clinician-Investigator with an interest in knowledge translation, including studies of novel ways to implement respiratory guidelines, and the use of electronic tools to bridge the clinical care gap.

## Pre-publication history

The pre-publication history for this paper can be accessed here:

http://www.biomedcentral.com/1472-6920/13/146/prepub

## Supplementary Material

Additional file 1 Contains: i) Post-Rotation Questionnaire; and ii) Questionnaire Scoring Scheme and Classification of Question Types. Click here for file
