# Supplementary material for: The effect of patient care order sets on medical resident education: a prospective before-after study
Source: BMC Med Educ. 2013 Nov 6;13:146. doi: 10.1186/1472-6920-13-146 (PMC3829666; doi:10.1186/1472-6920-13-146)
Supplement: Additional file 1 — Contains: i) Post-Rotation Questionnaire; and ii) Questionnaire Scoring Scheme and Classification of Question Types. [file 1472-6920-13-146-S1.docx]

Thank you for agreeing to complete this questionnaire. Your voluntary participation in this study will help us better understand the role of order sets in medical education and patient care. Please answer all questions to the best of your abilities.

**Part A: Demographic characteristics**

1) Age (years): [ ] <20 [ ] 20-24 [ ] 25-29 [ ] 30-34 [ ] 35-39 [ ] >40

2) Gender: [ ] M [ ] F

3) I am a resident in Training program: ___________ Year (e.g. R1): _____

4) a) Check the box that best describes your clinical experience in the past 1 month with a patient whose most responsible admission diagnosis was a COPD exacerbation:

[ ] I participated in writing the admission orders for a patient with a COPD exacerbation

[ ] I was involved in the care of a patient with a COPD exacerbation but did not write admission orders

[ ] I had no direct clinical exposure to a patient with a COPD exacerbation

b) If you did participate in writing admission orders for a patient with a COPD exacerbation, did you use the standardized COPD exacerbation admission order set?

[ ] Yes [ ] No

Please take a moment to consider your use of the order set over the last month, and think about how it affected your learning and your day-to-day practice. We would like to make sure that the order sets are helpful to resident learning and your comments are key to further improvements. Please answer all of the following questions, and include as many details as possible.

(c) If you did use the standardized COPD exacerbation admission order set:

(i) How did you use it?

[ ] Only to complete admission orders

[ ] As a reference for learning about COPD

[ ] Both to complete admission orders and as a reference for learning about COPD

(ii) Did you look up any of the information that was provided in the order set?

[ ] Yes [ ] No

(iii) How do you think it affected your *knowledge* of COPD and how did it do so?

[ ] Improved how: _________________________________________________

[ ] Deteriorated _____________________________________________________

[ ] No effect _____________________________________________________

(iv) How do you think it affected your *skill* in managing COPD and how did it do so?

[ ] Improved how: _________________________________________________

[ ] Deteriorated _____________________________________________________

[ ] No effect _____________________________________________________

5) a) Check the box that best describes your clinical experience in the past 1 month with a patient whose most responsible admission diagnosis was a CF exacerbation:

[ ] I participated in writing the admission orders for a patient with a CF exacerbation

[ ] I was involved in the care of a patient with a CF exacerbation but did not write admission orders

[ ] I had no direct clinical exposure to a patient with a CF exacerbation

b) If you did participate in writing admission orders for a patient with a CF exacerbation, did you use the standardized CF exacerbation admission order set?

[ ] Yes [ ] No

(c) If you did use the standardized CF exacerbation admission order set:

(i) How did you use it?

[ ] Only to complete admission orders

[ ] As a reference for learning about CF

[ ] Both to complete admission orders and as a reference for learning about COPD

(ii) Did you look up any of the information that was provided in the order set?

[ ] Yes [ ] No

(iii) How do you think it affected your *knowledge* of CF and how did it do so?

[ ] Improved how: _________________________________________________

[ ] Deteriorated _____________________________________________________

[ ] No effect _____________________________________________________

(iv) How do you think it affected your *skill* in managing CF and how did it do so?

[ ] Improved how: _________________________________________________

[ ] Deteriorated _____________________________________________________

[ ] No effect _____________________________________________________

**Part B: Admission order writing**

**Case 1:**

A 60-year-old current smoker of two packs-per-day with a 6-year history of COPD presents to the emergency room with a 3-day history of increasing dyspnea and an increase in the amount of sputum he is producing. His last exacerbation was 10 weeks ago, at which time he had completed a 7-day course of Levofloxacin. He has no known drug allergies. Physical exam reveals mild respiratory distress, pulse 92 regular, blood pressure 130/80, respiratory rate 16, oxygen saturation 94%. A chest x-ray shows hyperinflation and no acute infiltrates. An arterial blood gas is as follows: pH 7.29, paO_2_ 65 mmHg, pCO_2_ 50 mmHg. You have decided to admit this patient for an acute exacerbation of COPD.

Please write admission orders for this patient:

_________________________________________________________________

_________________________________________________________________

_________________________________________________________________

_________________________________________________________________

_________________________________________________________________

_________________________________________________________________

_________________________________________________________________

_________________________________________________________________

_________________________________________________________________

_________________________________________________________________

_________________________________________________________________

_________________________________________________________________

_________________________________________________________________

_________________________________________________________________

_________________________________________________________________

_________________________________________________________________

_________________________________________________________________

_________________________________________________________________

_________________________________________________________________

_________________________________________________________________

_________________________________________________________________

_________________________________________________________________

_________________________________________________________________

_________________________________________________________________

_________________________________________________________________

_________________________________________________________________

_________________________________________________________________

_________________________________________________________________

_________________________________________________________________

_________________________________________________________________

_________________________________________________________________

_________________________________________________________________

_________________________________________________________________

_________________________________________________________________

_________________________________________________________________

**Case 2:**

A 21-year-old woman with cystic fibrosis presents to her out-patient appointment with a 3-week history of increasing cough and sputum, for which she has been on Septra, with no improvement. She has been increasingly short of breath with thicker, darker sputum over the past week and she has lost 5 lbs in the past week. She has missed two days of work. Despite not eating well, she has noted blood sugars of 11-12. You review her chart and learn that she is “Cepacia positive” and also grows Pseudomonas aeruginosa. Her other medications include Salbutamol 1 puff inhaled bid, Tobramycin 160 mg nebs bid, Pulmozyme 2.5 mg nebs daily, Cotazym ECS 8 with each meal, ADEK 1 tablet once daily, Losec 20 mg once daily. On review of systems, she reports post-prandial nausea and constipation. She has no known allergies. On examination, there are expiratory wheezes throughout. You have decided to admit this patient for a CF exacerbation.

Please write admission orders for this patient:

_________________________________________________________________

_________________________________________________________________

_________________________________________________________________

_________________________________________________________________

_________________________________________________________________

_________________________________________________________________

_________________________________________________________________

_________________________________________________________________

_________________________________________________________________

_________________________________________________________________

_________________________________________________________________

_________________________________________________________________

_________________________________________________________________

_________________________________________________________________

_________________________________________________________________

_________________________________________________________________

_________________________________________________________________

_________________________________________________________________

_________________________________________________________________

_________________________________________________________________

_________________________________________________________________

_________________________________________________________________

_________________________________________________________________

_________________________________________________________________

_________________________________________________________________

_________________________________________________________________

_________________________________________________________________

_________________________________________________________________

_________________________________________________________________

_________________________________________________________________

_________________________________________________________________

_________________________________________________________________

_________________________________________________________________

_________________________________________________________________

_________________________________________________________________

_________________________________________________________________

Please hand in Part A and Part B.

You will then receive Part C.

Make sure that you have filled out the top right corner of EACH page.**Part C: Short Answer Questions**

**Inpatient management of chronic obstructive pulmonary disease (COPD):**

1. List 2 differential diagnoses in a patient presenting with an acute exacerbation of COPD.

i) _________________________________________________________________

ii) _________________________________________________________________

1. List 3 criteria for hospitalization of a patient with an acute exacerbation of COPD?

i) _________________________________________________________________

ii) _________________________________________________________________

iii) _________________________________________________________________

1. a) What percent of exacerbations of COPD are due to respiratory infections?

0-19% 20-39% 40-59% 60-79% 80-100%

b) List 2 bacterial organisms responsible for precipitating an acute exacerbation of COPD.

i) _________________________________________________________________

ii) _________________________________________________________________

c) List 4 non-bacterial organisms typically responsible for precipitating an acute exacerbation of COPD.

i) _________________________________________________________________

ii) _________________________________________________________________

iii) _________________________________________________________________

iv) _________________________________________________________________

1. a) What are the 3 main criteria used to determine whether or not to use antibiotics in the treatment of an acute exacerbation of COPD (i.e. without pneumonia)?

i) _________________________________________________________________

ii) _________________________________________________________________

iii) _________________________________________________________________

b) How many of these criteria are required to justify antibiotic use for an acute exacerbation of COPD? ___________________________________________________________

1. If your patient has a history of congestive heart failure, what implication does this have on your management plan?

___________________________________________________________________

1. if your patient has an FEV1 < 50%, what 2 implications does this have on your management plan?

i) _________________________________________________________________

ii) _________________________________________________________________

1. Other than drug allergy or use of similar antibiotic class in the last 3 months, list one reason why you would choose:

a) Levofloxacin over Azithromycin

___________________________________________________________________

b) Clavulin over Septra

___________________________________________________________________

c) Ciprofloxacin over Clavulin

___________________________________________________________________

d) Ceftazidime over Doxycycline

___________________________________________________________________

1. a) What are the criteria for early use of non-invasive positive pressure ventilation (NIPPV) in patients with COPD exacerbations?

___________________________________________________________________

b) What type of NIPPV is recommended in these patients?

___________________________________________________________________

c) What patient outcomes have been shown to improve with early use of NIPPV when appropriate?

i) _________________________________________________________________

ii) _________________________________________________________________

1. What are 2 implications for management if a patient has had antibiotics in the last 3 months?

i) _________________________________________________________________

ii) _________________________________________________________________

1. What is the single most important intervention to preserve lung function?

____________________________________________________________________

1. What 2 recommendations might you suggest to your patient that may decrease his/her risk of getting another exacerbation?

i) _________________________________________________________________

ii) _________________________________________________________________

1. Circle “True” or “False” after each of the following statements:

A “COPD action plan” (in which patients are instructed on how to recognize an exacerbation and self-manage) has been demonstrated to:

a) result in earlier initiation of antibiotics True False

b) decrease healthcare utilization True False

c) improve quality of life True False

**Inpatient management of cystic fibrosis (CF):**

1. What are features of the infection control policy on the ward that must be communicated to all CF patients at the time of admission?

i) _________________________________________________________________

ii) _________________________________________________________________

iii) _________________________________________________________________

1. List 3 antibiotics that have some activity against Burkholderia cepacia complex.

i) _________________________________________________________________

ii) _________________________________________________________________

iii) _________________________________________________________________

1. a) What are the indications for intravenous antibiotics in patients with a CF exacerbation?

i) _________________________________________________________________

ii) _________________________________________________________________

iii) _________________________________________________________________

- 1. When using intravenous Tobramycin, what parameter should be monitored?

____________________________________________________________________

- 1. At what frequency or time interval should this parameter be checked?

____________________________________________________________________

1. a) What is the indication for inhaled antibiotics in patients with a CF exacerbation?

___________________________________________________________________

b) List 2 inhaled antibiotics that can be used in the treatment of CF exacerbation.

i) _________________________________________________________________

ii) _________________________________________________________________

1. a) What are two indications for inhaled anti-inflammatory medications in patients with a CF exacerbation?

i) _________________________________________________________________

ii) _________________________________________________________________

b) List two inhaled anti-inflammatory medications that can be used in patients with CF.

i) _________________________________________________________________

ii) _________________________________________________________________

1. a) When is non-invasive positive pressure ventilation indicated in patients with a CF exacerbation?

___________________________________________________________________

b) What mode of non-invasive positive pressure ventilation is recommended in patients with CF (at baseline or during an acute exacerbation)?

___________________________________________________________________

1. List two endocrine/metabolic consequences of CF.

i) _________________________________________________________________

ii) _________________________________________________________________

1. List 4 gastrointestinal consequences of CF, as well as one pharmacologic agent that can be used to treat it in patients with CF.

i) _________________________________________________________________

treatment ________________________________________________________

ii) _________________________________________________________________

treatment ________________________________________________________

iii) _________________________________________________________________

treatment ________________________________________________________

iv) _________________________________________________________________

treatment ________________________________________________________

| **Question** | Type 1^a^ | **Type 2^b^** | **Type 3^c^** | **Total** |
| --- | --- | --- | --- | --- |
| **COPD questionnaire** | | | | |
| 1 |  | 1 point |  |  |
| 2 |  |  | 3 points |  |
| 3a |  |  | 1 point |  |
| 3b | 2 points |  |  |  |
| 3c | 2 points |  | 2 points |  |
| 4a | 1 ½ points |  |  |  |
| 4b | ½ point |  |  |  |
| 5 |  | 1 point |  |  |
| 6 |  | 2 points |  |  |
| 7 |  | 4 points |  |  |
| 8a | ½ point |  |  |  |
| 8b | ½ point |  |  |  |
| 8c |  |  | 2 points |  |
| 9 |  | 2 points |  |  |
| 10 | 1 point |  |  |  |
| 11 | 3 points |  |  |  |
| 12 |  |  | 3 points |  |
| Subtotal points | 11 points | 10 points | 11 points | 32 points |
|  | | | | |
| CF questionnaire | | | | |
| 1 | 3 points |  |  |  |
| 2 |  |  | 3 points |  |
| 3a |  |  | 3 points |  |
| 3b | 1 point* |  | 1 point* |  |
| 3c | 1 point* |  | 1 point* |  |
| 4a |  |  | 1 point |  |
| 4b | 2 points |  |  |  |
| 5a |  |  | 2 points |  |
| 5b | 2 points |  |  |  |
| 6a |  |  | 1 point |  |
| 6b | 1 point |  |  |  |
| 7 |  | 2 points |  |  |
| 8 |  | 8 points | Depending on answer |  |
| Subtotal points | 10 points | 10 points | 10 points | 30 points |
|  | | | | |
| Total points | 21 points | 20 points | 21 points | 62 points |

*Depending on the answer provided by the trainee, the question was scored as either a type 1 or type 3 question.

**^a^ Type 1 question:** Knowledge transmitted directly by order set evidence-based prompts (for example, the COPD order set explicitly listed criteria for early use of non-invasive positive pressure ventilation; a test question asking for these criteria was classified as a type 1 question)

**^b^ Type 2 question:** Other knowledge conferred through order set use (for example, the CF order set included a section listing “GI medications” such as omeprazole, ranitidine, domperidone, ursodeoxycholic acid, polyethylene glycol and mineral oil; a test question asking for gastrointestinal consequences of cystic fibrosis and a corresponding pharmacotherapeutic agent for each was classified as a type 2 question)

**^c^ Type 3 question:** Knowledge that could not be gained through order set use (for example, a question asking what percent of exacerbations of COPD are due to respiratory infections was classified as a type 3 question, as this content was not included in the order set) (question types 1,2, and 3) (Online Appendix 2)
